# Integrating Early-Stage Drug Development with Clinical Networks; Challenges and Opportunities: The City of Hope Developing Experience

**DOI:** 10.3390/jcm12124061

**Published:** 2023-06-15

**Authors:** Miguel A. Villalona-Calero, Jyoti Malhotra, Vincent Chung, Yan Xing, Stacy W. Gray, Heather Hampel, Stephen Gruber, Kevin McDonnell

**Affiliations:** City of Hope National Medical Center, Department of Medical Oncology, Duarte, CA 91010, USA

**Keywords:** integration, community clinical network, phase 1, genomic-driven, clinical trials

## Abstract

Recent data suggest that patients with advanced cancer who participate in biomarker/genomically informed early-stage clinical trials experience clinical benefit. While most early-stage clinical trials are conducted in major academic centers, the majority of cancer patients in the United States are treated in community practices. Here, we describe ongoing efforts at the City of Hope Cancer Center to integrate our network community oncology clinical practices into our academic, centralized biomarker/genomic-driven, early-stage clinical trial program to build an understanding of the approaches that provide the benefits of early-stage clinical trial participation to community patients. Our efforts include three key initiatives: the development of a virtual “Refractory Disease” phase 1 trial matching televideo clinic, the construction of infrastructure to support the expansion of phase 1 clinical trials to a distant regional clinical satellite hub, and the implementation of an enterprise-wide precision medicine, germline, and somatic testing program. Our work at City of Hope may serve as an example to facilitate similar efforts at other institutions.

## 1. Introduction

Clinical development of a new molecular or biological entity is a long and costly process [1,2]. Advances in science and technology have enabled academic centers, biotechnology companies, and major pharmaceutical companies to access an ever-increasing number of agents with sufficient therapeutic potential to test in the clinic [3,4,5]. However, many drugs fail during drug development, either because of unacceptable toxicity, or lack of target effect [6,7]. Properly designed, executed, and analyzed early-stage clinical trials are fundamental for a new drug or combination of drugs to obtain eventual approval for marketing.

The complexity of performing early-stage trials and the burden posed to patients in terms of time, frequent travel, required procedures, and traditionally low response rates impacts referral patterns, and has historically limited the access of most patients to new anticancer agents until much later stages in the process of drug development [8,9]. Major academic centers and a few selected community practices currently share the majority of the responsibility for conducting these trials.

Clinicians as well as non-clinicians may question whether it is worthwhile for patients treated in community practices to pursue clinical trials with drugs being tested in early clinical drug development. Previous work in the field arguing against or encouraging referrals and participation is limited. Decoster et al. conducted a review of the antitumor activity and toxic deaths reported in single-agent phase I clinical trials in cancer patients using cytotoxic compounds between 1972 and 1987 [10]. A total of 6639 patients were accrued to 211 trials studying 87 compounds. There were 23 (0.3%) complete responders and 279 (4.2%) partial responders for an overall response rate (RR) of 4.5% among all entries. Toxic deaths were rare and reported in only 31 patients (0.5% of the entire population). Similarly, Von Hoff et al., in 1991, reported their review of 228 phase 1 trials over a period of 14 years [11]. There were 75 complete and 432 partial responses recorded among 7960 patients for an overall objective RR of 6%.

In contrast, Chihara et al. reported on National Cancer Institute (NCI) sponsored phase 1 trials conducted between 2000 and 2019 [12]. The overall RR for all trials during the study period was 12.2% among 9325 patients and the complete RR was 2.7%. Overall response increased from 9.6% during the period 2000 to 2005 to 18% between 2013 and 2019, and complete RR from 2.5% to 4.3%. Overall RR for combination therapy was substantially higher than for monotherapy (15.8% vs. 3.5%). Furthermore, Chakiba et al. conducted a literature review of 224 phase 1 trials that were published from 1 January 2014 to 30 June 2015 [13]. The overall RR was 19.8%. Phase 1 trials employing an enrichment design (i.e., specific histologic characteristics, a specific biomarker, or both) were associated with a higher probability of clinical benefit, and a higher probability of an objective tumor response occurred among patients enrolled in phase 1 trials that included expansion cohorts.

Additional studies evaluated the impact of biomarker treatment strategies compared to an “all comers” approach in clinical trials. These analyses are limited in the phase 1 setting. Schwaederle et al. conducted a meta-analysis comparing patient outcomes in phase 1 studies that used a biomarker selection strategy with those that did not [14]. The analysis included trials performed between January 2011 and December 2013 and evaluated RR and progression-free survival (PFS). A total of 346 studies met the criteria for evaluation; 13,203 patients were treated within 351 study arms. Of these, 117 arms used a cytotoxic agent, whereas 234 arms used a targeted agent, with 57 (24.4%) being personalized. Non-personalized targeted agent arms had outcomes comparable with those that tested a cytotoxic agent. However, personalized arms using a genomic biomarker had a significantly higher median RR, 30% vs. 4.9% in the other arms, and a longer PFS, 5.7 months vs. 2.95 in the other arms. Furthermore, Mackley et al. [15] analyzed reports of 158 phase 1 trials published between January 2015 and July 2018; thus, not overlapping with the studies analyzed by Schwaederle and collaborators. The studies involved 6707 patients. The combined RR was 4%. Among the trials using tumor biomarkers as the eligibility criteria, the RR was higher: 12% vs. 4.9%. However, the same was true of trials focusing on single tumor type (13%) compared to multiple tumor types (3.8%). There were no treatment-related deaths, but the proportion of grade 3 to 4 toxicity was 13.2%.

Von Hoff et al. conducted a pilot study using molecular profiling (MP) of patients’ tumors to find potential targets and select treatment based on these findings [16]. This group evaluated the premise that a substantial group of patients selected by this approach would experience improved clinical outcomes compared to their outcome with the immediate priorly administered treatment. The null hypothesis of ≤15% of this patient population having a PFS on MP-selected therapy/PFS on prior therapy of ≥1.3 was rejected. Eighteen of sixty-six patients (27%) had a PFS ratio of ≥1.3.

Important caveats in analyses of the clinical benefit of phase 1 trials in cancer patients, molecularly driven or not, include the multiplicity of tumor histological types usually accrued and the required multiple-dose evaluation steps for safety evaluation and regulatory agency mandates. Thus, even when a target is identified and there is an agent reasonably expected to result in efficacy, the range of doses tested include some below target inhibition and some unnecessarily toxic, beyond the requirement for target inhibition. Importantly, in order for a biological target to be clinically relevant and a molecularly targeted approach beneficial, potent drugs should be available that can interact successfully with the target without significant off-target toxicity. 

Thus, overall, it is reasonable to conclude that it is worthwhile for an academic center to pursue systematic rational efforts to obtain promising targeted or immune-interacting agents, and to provide increased access to patients and care providers in community practices of these agents at the time when patients need them the most. That is, to provide these agents when their disease has become refractory to the available standard of care therapeutic approaches.

## 2. Current Challenges for Early-Stage Clinical Trials

Although the numbers of clinical trials with novel agents have increased, and expansion cohorts have become the routine, the number of clinical sites involved in a typical industry-sponsored clinical trial have exponentially increased, limiting the slots available to individual sites. This practice comports with corporate mandates to fill out available slots as quickly as possible in order to decrease the time to completion of the clinical trial. 

Safety issues arising from the coordination of multiple sites have been partially offset by the institution of frequent investigators’ calls and virtual meetings. However, these meetings often involve multiple time zones, substantial time demands and incomplete or stale data sets that may frustrate participants and result in attendee attrition. Unintentionally, these circumstances create competition among the sites for patients’ slots, resulting in insufficient slots for patients who have time-sensitive needs for these investigational therapeutics. 

From the participating sites’ perspective, challenges include insufficient staff recruitment and retention, low capacity of treating units and hospitals (such as during the SARS-CoV-2 pandemic) [17,18], increasing clinical and regulatory demands on the clinicians–investigators’ time, as well as prolonged time to activation of trials, frequent amendments, and reporting requirements [19]. Tracking down the genomic analyses of referred patients among the array of different analytic platforms not integrated into the medical records has also proven to be a difficult task. 

From the community oncology perspective, and impacting community sites integration into clinical trials, referring patients to be placed on phase 1 waiting lists is, at a minimum, inconvenient and impractical. Furthermore, the necessity for patients to travel long distances for very frequent clinic visits, the requirement in many cases for tumor sample prescreening with uncertain outcome, together with multiple patients’ procedures, and imaging while on trial, dampen the enthusiasm of previously motivated patients and families. An additional, sometimes insurmountable, challenge to community patients’ participation is the approval process and financial limits of health management organizations (HMOs) [20].

## 3. City of Hope (COH) Community Oncology Practice Network

**Figure 1:**
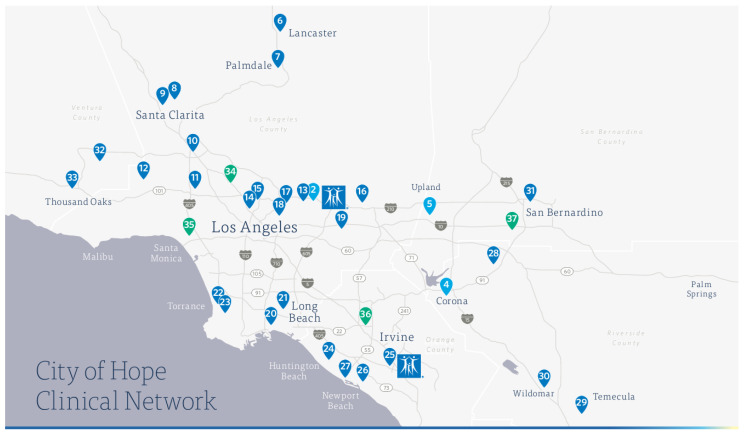
COH community practice sites.

The COH Clinical Practice Network serves populations located in four Southern California counties: Los Angeles, Orange, San Bernardino/Inland Empire, and Riverside, encompassing 33,109 square miles and approximately 18 million residents (Figure 1). These sites were selected due to their demonstrated telehealth access, strong leadership commitment to quality care and research, and the diverse populations served. Over 30 community satellite practice sites with >150 physicians are distributed throughout our catchment area. The catchment area is one of the most diverse regions in the country (45% Hispanic/LatinX; 12% Asian/Pacific Islander; 6% Non-Hispanic Blacks) and includes a clinical practice site serving a low resource and socio-economically disadvantaged population in the California high-desert region. The majority of sites provide multidisciplinary cancer care. We share unique utilization of the EPIC electronic medical record, employment of COH tailored “Via” pathways, disease-focused tumor registries, as well as precision medicine genomics evaluation. Twenty percent of Duarte campus referrals for complex care or unique studies come from our community satellite practices. A Clinical Outpatient 190,000 Sq. Ft. hub capable of conducting all stages of clinical trials (Lennar Foundation Cancer Center) recently opened in Irvine, Orange County.

The COH clinical practice network provides access to a very large cancer patient population (October 2020–September 2021: 104,378 unique patients; 359,679 completed appointments); (October 2021–September 2022: 107,870 unique patients; 367,812 completed appointments). The patients served are spread throughout a very large geographical territory and the network practices have different capabilities. Thus, concerted efforts are needed if we are to provide on-site access or directed channeling of patients to early-stage clinical investigations when appropriate. 

## 4. Clinical Research Integration Opportunities

A number of initiatives have enhanced early-stage clinical investigation at COH. These include rapid clinical trial activation times (<90 days on average); uniform protocol templates; a single application form used by the protocol review committee, data safety monitoring board, and institutional review board; upstaffed regulatory start-up and contracting teams; specialized teams to build Epic Beacon and OnCore content; increased use of Master Clinical Trial Agreements with biotech and pharmaceutical companies that reduces the involvement of the general counsel; and participation as a leading academic organization in the NCI Early Therapeutics Clinical Trials Network. 

Through an institution-sponsored Precision Medicine program, tumor whole exome and transcriptome sequencing together with germline gene panel sequencing are performed for City of Hope patients and their families. A digital infrastructure and informatics platform provides logistical and analytic support. 

## 5. Ongoing COH Phase 1 Program Integrative Initiatives

### 5.1. Development of a Virtual “Refractory Disease” Phase 1 Trial—Matching Tele-Medicine Oncology Clinic

During this televideo-medicine-enabled clinic, interested patients referred by COH oncologists (community and academic) or regional HMO oncologists are evaluated. Referred patients have incurable tumors for which standard effective conventional therapies have been exhausted or are non-existent. 

A full-time dedicated coordinator oversees the preparation of the clinic ahead of the scheduled appointment. The coordinator contacts referring physicians and patients and retrieves medical records which are made available to the phase 1 oncologist prior to the visit. The retrieval of all prior pathology data and reports and tumor genomic analyses is particularly challenging. In our experience, outside, often diverse, genomic analyses are frequently not incorporated into electronic medical records. If targetable alterations are identified during review, patients will be offered targeted treatment with therapies tailored to their tumor biology if not previously performed. In addition, in some cases, historical genomic analyses provide clues to potential targets amenable to not yet approved investigational agents when biological rationale exists supporting potential efficacy. If no such mutations are found (or while waiting for testing), available non-target specific phase 1 trials, such as those with novel mechanisms of action or immune-system-targeted therapies, are discussed. If the patient agrees to participate in a trial and they meet preliminary eligibility criteria during the virtual visit screening, the patients will visit the COH Duarte campus (or the Orange County clinical-research Hub, see below) to undergo examination and consent to the particular study; subsequently, they receive screening for trial initiation. However, if a tumor-specific later stage trial is available that better fits the patient, a recommendation and referral to the proper tumor-specific physician(s) is made, after obtaining approval from the referring physician. 

These clinics (once a week with rotating phase 1 oncologists) were initiated during 2022 with great acceptance from patients and medical providers alike. Feedback communication with referring physician within 48 h is routinely performed. Patients are also provided access to our centralized germline and somatic genomic testing, if not previously performed (see below). 

### 5.2. Expansion of Phase 1 Trials to City of Hope—Orange County

The large community oncology practice network at COH has increased our catchment area, enabled the enrollment of patients from diverse ethnicities and backgrounds as well as providing the patients the opportunity to seek specialized clinical care closer to home. However, early-phase trials can only be opened at sites that have the capabilities to conduct these trials. These requirements include the handling of research samples that may involve frequent and long hours of pharmacokinetic sampling and specialized on-site research pharmacy and radiology. Patients identified through the COH community network eligible for early-phase trials still must travel to the main campus in Duarte multiple times a month. 

One recent development in the COH enterprise has been the opening of the City of Hope Orange County Lennar Foundation Cancer Center in Irvine, Orange County, in August 2022.

**Figure 2:**
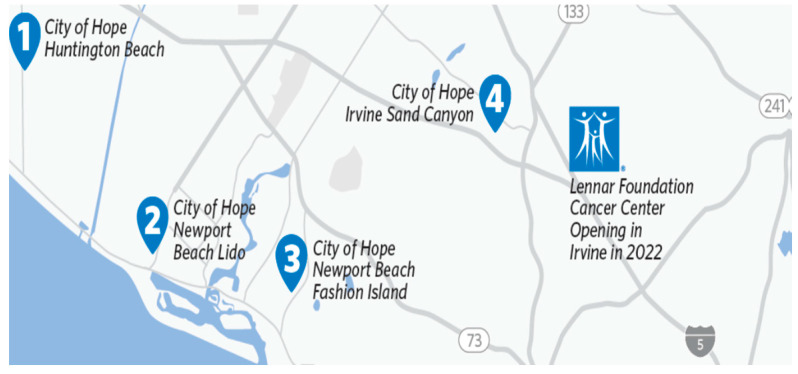
Map of COH Orange County clinical sites.

The Orange County Cancer Center offers all comprehensive cancer services with a dedicated clinical research unit and allows the conduct of on-site early-phase cancer trials. Within the County, this cancer center serves as a ‘hub’ offering a full array of clinical operations serving as the ‘spokes’ or regional community sites within Orange County. These ‘spoke’ sites typically provide only clinical services and late-phase trials. As the new cancer center is located within 25 miles from each of these regional sites (Figure 2), patients can be easily routed to the hub for early-phase trials as well as more specialized services. 

Adding early-phase trials to a second campus beyond the academic Duarte campus created a number of challenges: increased regulatory processing, the establishment of an efficient clinical workflow for each trial, and the coordination of Orange County laboratories with laboratories at the Duarte campus for sample processing and shipping. Dedicated efforts have succeeded in ensuring that most services required for conducting early-phase trials are now operational at the Orange County site, such as contracting, institutional board review, data safety monitoring, budget review, and an electronic health system as well as phase I disease team management.

### 5.3. Precision Medicine Network Initiative

The efficient and successful conduct of early-stage drug trials depends critically upon the investigators’ ability to identify patients whose tumor mutational profiles match the molecular target of an investigational drug [21,22,23,24]. Limiting the success of early-stage drug trials, patients frequently experience difficulty in obtaining tumor sequencing, trialists often lack ready access to completed studies, and clinicians may experience challenges in interpreting the often disparate, dense, and abstruse tumor sequencing reports [25,26,27,28,29]. These limitations may compound in the community oncology setting due to inconsistent tumor sequencing practices, inadequate administrative structure, and lack of advanced molecular expertise [30]. Helping to ameliorate these limitations and accelerate the progress of early-stage drug trials for its community oncology practice partners, City of Hope created the Center for Precision Medicine.

## 6. The City of Hope Center for Precision Medicine

The advent of targeted cancer therapies ushered in the era of precision medicine in clinical oncology [31]. City of Hope (COH), recognizing the unparalleled promise of precision medicine, established the COH Center for Precision Medicine (COH-CPM) in 2020 [32]. The COH-CPM aims to harness genomic-driven insights to pioneer personalized prevention and treatments towards improving the outcomes and quality of life for patients and their families. To accomplish its mission, the COH-CPM initiated the INSPIRE (Implementing Next-generation Sequencing for Precision Intervention and Risk Evaluation) study, a universal access investigation open to all patients at COH with a personal and/or family history of cancer. 

COH-INSPIRE participants receive germline genetic assessment through testing with a custom 155 cancer gene panel [33]. Tumors of patients with an available cancer specimen undergo somatic tumor-normal whole exome and whole transcriptome sequencing [34,35]. To conduct the INSPIRE study, COH-CPM relies on an expert team of enrollment specialists, genetic counselors, and cancer genetic physicians who facilitate the participation and clinical management of patients. Since its inauguration, the INSPIRE study has experienced tremendous success with enrollment of nearly 15,000 patients. INSPIRE patients whose sequencing results pose complex genetic, genomic, and clinical questions receive in-depth review at 2 weekly clinical case conferences: a Genetics Case Conference and a Precision Oncology Tumor Board (POTB). 

### 6.1. COH-CPM Clinical Case Conferences

Genetic counselors and cancer genetics physicians conduct the Genetics Case Conference with the aim of resolving challenging problems related to germline findings and genetic risk. The INSPIRE study has observed that nearly 1 in 5 (2654/14,346 [18.5%]) patients carry a germline pathogenic variant requiring clinical review and management; this significantly elevated pathogenicity rate ensures a full volume of complex case reviews but also, more clinically significant, validates a universal access model of precision medicine availability. 

### 6.2. Precision Oncology Tumor Board

The second weekly conference, the POTB, complements the Genetics Case Conference. The POTB provides interpretation, targeted therapeutic insights, and clinical trial eligibility information related to tumor whole exome and transcriptome sequencing results. The POTB enlists the expertise of a multidisciplinary team comprising, among others, medical and surgical oncologists, genomic scientists, genetic counselors, and computational biologists. To date, the POTB has completed over 150 deep-dive analyses to help the tangible delivery of precision medicine to COH patients, positively impacting their treatment, health, and well-being. 

## 7. Centralized Logistical Operation of COH-INSPIRE

Inaugural COH-CPM INSPIRE activities focused on optimizing precision medicine operations at the central, academic COH Duarte campus. Initial efforts sought to design, implement, and iteratively improve four core precision medicine operations: patient enrollment, specimen processing, germline and somatic tumor next-generation sequencing, and clinical management. Most patients enroll in the INSPIRE study through assigned study consenters stationed in the oncology subspecialty clinic where they receive treatment. Consenters may also enroll a patient remotely via a televideo protocol should in-person consenting prove infeasible. For germline DNA assessment, patients provide blood samples to a central specimen collection laboratory situated on campus. The COH Pathology department assumes responsibility for retrieving fresh-frozen, paraffin-embedded tumor blocks for somatic sequencing. Furthermore, the COH Pathology department oversees delivery of the germline and somatic tumor specimens to commercial NGS vendors who perform CLIA/CAP-grade germline panel and somatic whole exome and transcriptome sequencing. 

Vendors typically complete the sequencing of specimens within 10–14 days of specimen receipt and deliver test reports directly to the COH electronic medical record as primary BAM, FASTQ, and VCF sequencing files. A centralized electronic data warehouse, POSEIDON, receives copies of the primary BAM, FASTQ and VCF sequencing files [36]. Data analysts and computational biologists have access to these data files for downstream analysis. A clinical team of genetic counselors and cancer genetic physicians review all sequencing results; this team identifies patients requiring clinical management and/or further in-depth analyses. Optimization of these core operational activities has enabled high volume patient participation in the INSPIRE study at the COH Duarte campus. 

## 8. Expansion of INSPIRE to the COH Community Oncology Network

Iterative improvements in the INSPIRE protocol established an efficiently functioning, fully interoperable, and incrementally more agile precision medicine workflow. Since initial optimization, COH-CPM has continued expansion of INSPIRE with serial introduction of the study across COH community oncology practices. To achieve streamlined integration of INSPIRE across the community practice enterprise, COH-CPM adopted a “hub-and-spoke” mode of operational logistics. Administration and pathology processing operations remain anchored at the Duarte campus hub, while specimen collection and in situ INSPIRE consenting takes place nodally at the community oncology clinic spokes. As with INSPIRE cases originating at the Duarte campus, all community oncology INSPIRE cases qualify for review through the Genetics Case Conference and POTB. Precision Medicine teams organize and conduct reviews at the central Duarte campus with remote televideo participation of community practices.

In 2021, COH-CPM successfully commenced network community INSPIRE participation. Among the first community oncology sites to participate, the COH Upland clinic, located in San Bernadino County, approximately 30 miles east of the central Duarte campus, serves a racially and ethnically diverse, historically underserved patient population. To date, over 1000 Upland community oncology patients have completed germline and somatic sequencing through the INSPIRE study. To expedite timely and convenient genetics care at the Upland clinic, a cancer genetics-trained surgical oncologist provides in-person services to INSPIRE patients requiring ancillary management. 

COH-CPM has recently expanded INSPIRE to 20 COH community oncology practices; ongoing expansion efforts continue towards achieving full participation of all COH community oncology practices. Full expansion of INSPIRE promises the diverse community oncology patient population not only facilitated access to precision medicine resources but also widened avenues for potentially clinically impactful early drug development participation. 

## 9. Leveraging INSPIRE to Accelerate Early Drug Development across the COH Community Oncology Network

Over the past 2 decades, the number of clinical trials requiring genomically informed biomarker qualification has increased exponentially from 15% to greater than 50% [37,38]. Often utilized qualifying biomarkers include, among others, germline and somatic pathogenic genetic variants and fusions, tumor mutational burden, homologous recombination deficiency, microsatellite instability, checkpoint inhibitor protein expression, and hormone receptor states [39]. INSPIRE directly or secondarily (through downstream analyses) has the ability to assess the gamut of these biomarkers; moreover, standard INSPIRE review protocols identify patients eligible for clinical trial enrollment based upon their genomic biomarker profile. These embedded review protocols permit proficient, routine identification of clinical trial-eligible patients and undergird a high-volume clinical trial selection process. 

Expansion of INSPIRE across the COH community network affords the early drug development program four transformational opportunities to 1—increase early-stage clinical trial enrollment; 2—optimize investigational drug and clinical trial matching; 3—improve drug response rates; and 4—enhance healthcare equity for COH oncology community participants. 

The INSPIRE study promotes not only improved quantity of enrollment, but also higher quality. INSPIRE’s comprehensive assessment of germline, somatic whole exome and transcriptome alterations allows more specific drug matching and, consequently, more precise enrollment into early-stage drug development trials. More precise enrollment and optimized drug matching predicts improved drug response rates and, consequently, accelerated drug development [40,41]. Conversely, failure to “select the right drug for the right patient” may result in a lack of therapeutic efficacy and abandonment of further drug development efforts [42,43,44].

Historically underserved populations frequently demonstrate compromised awareness of genetics and genomics and the impact that these areas of medicine may have on their health and oncology treatment options [45,46]. The INSPIRE study proactively enrolls community oncology practice patients, many of whom present from marginalized and underserved regions of the COH catchment area. INSPIRE enrollment specialists and genetic counselors directly engage these patients, explaining the rationale and process of genetic and genomic testing; subsequently, patients meet with genetic counselors and cancer genetics physicians to review their test results and discuss health implications for them and their families. These direct interactions often represent the patients’ first awareness of precision medicine and its promises. In tandem with increasing awareness, INSPIRE provides concrete access to the resources of precision medicine testing and offers a navigable clinical pathway to enter early-stage drug development studies [47].

Community participation in the INSPIRE study promotes patient health agency and autonomy. Knowledge of individual genetic alterations, both germline and somatic, empowers patients to make informed decisions regarding therapeutic options as well as clinical trial participation. Informed engagement with genetic specialists and early-stage drug development trialist moves the patient closer to the ideal of authentic shared decision-making in their cancer care [48,49].

## 10. Conclusions

Overcoming several significant practical challenges, our ongoing initiatives at COH have allowed our community clinical network to leverage the ever-transforming landscape of genetics, genomic, and novel therapies that is impacting cancer care. Our program has achieved excellent clinician and patient satisfaction. To date, the program has performed >12,000 germline and >6000 somatic tumor unique patient tests for a diverse patient population comprising 47% Hispanics/Latinx, 29% API, 12% African Americans, and 1% Native Americans. 

Precision medicine-based innovation and discovery provide new opportunities for the early-stage drug development bench to clinic translational programs. In turn, these programs offer the COH community oncology patient population access to promising treatments.

We believe that the COH experience of developing and implementing a hub and spoke, early drug development program can serve as a model for other community oncology practices nationwide. Only with community integration can novel therapeutic discoveries reach their true clinical potential. However, such integration requires careful consideration and thoughtful deliberation regarding value versus cost, understanding not only therapeutic dividends but also the societal and ethical benefits of providing advanced genomic oncology care to underserved, disadvantaged populations. 

## Data Availability

Not applicable.
